# Targeting the apical domain of the transferrin receptor: Development of a new protein scaffold for cellular delivery

**DOI:** 10.1002/pro.70384

**Published:** 2025-11-18

**Authors:** Anuthariq Alikkam Veetil, Dick J. Sjöström, Cristian Iribarren, Camilla Mohlin, Elena Ambrosetti, Sinisa Bjelic

**Affiliations:** ^1^ Department of Chemistry and Biomedical Sciences Linnaeus University Kalmar Sweden; ^2^ Department of Medical Biochemistry and Biophysics Karolinska Institutet Stockholm Sweden

**Keywords:** flow cytometry, protein design, protein–protein interface, transferrin receptor, yeast surface display

## Abstract

Human transferrin receptor 1 (TfR) is essential for cellular iron homeostasis by internalizing the iron carrier proteins transferrin and ferritin. It is also an entry point for various pathogens, such as South American hemorrhagic fever caused by arenaviruses and the malaria parasite *Plasmodium vivax*, which utilize TfR to gain access to cells. The receptor is additionally upregulated in many aggressive cancers and at the blood–brain barrier. Altogether, the TfR is a highly relevant target for many medical applications, and novel protein‐interacting partners are sought after. A protein design strategy was explored here to develop a small protein that can be used for drug delivery across cell membranes, to investigate blood–brain barrier crossing, study endocytosis, or to block pathogen access to the apical domain. A computationally docked library of small protein scaffolds to the TfR apical domain, the native binding site of the *Machupo* arenavirus, was a starting point for the design and optimization. The best variants were expressed in a yeast surface display system and assessed for interaction with TfR by flow cytometry. One protein variant, which initially showed a low binding signal, was further optimized by directed evolution to bind to the target receptor at nanomolar concentration. The evolved construct, tagged with the enhanced green fluorescent protein (eGFP) and bacterially expressed, showed uptake similar to that of FITC‐coupled transferrin in a cell‐based assay. The designed protein can be utilized as a tool to target cell entry via TfR for drug delivery applications or as a foundation for developing antiviral therapeutics against arenaviruses.

## 1 INTRODUCTION

Human transferrin receptor 1 (TfR), a well‐characterized system, is responsible for iron homeostasis in cells with the iron protein carrier transferrin (Tf) (Andrews & Schmidt, 2007; Eckenroth et al., 2011; Lawrence et al., 1999; Testi et al., 2019). The Tf–TfR complex is internalized by clathrin‐mediated endocytosis, enabling intracellular release and supply of iron ions (Andrews & Schmidt, 2007). The receptor is ubiquitously present on cells; however, it is expressed at higher levels in many cancer types, as well as in the endothelial cells of the blood–brain barrier (Daniels et al., 2012; Ponka & Lok, 1999), which makes it a potential target for several therapeutic strategies. Structurally, TfR is a homodimeric (170 kDa), transmembrane glycoprotein with a large extracellular part composed of three domains: the apical, helical, and protease‐like domains (Lawrence et al., 1999). In addition to Tf, several endogenous proteins interact with the receptor, for example, the iron regulatory hemochromatosis protein, HFE, that binds to the partly overlapping Tf binding site on the helical and protease‐like domains (Bennett et al., 2000), and the iron carrier protein, ferritin, that binds to the TfR apical domain (Montemiglio et al., 2019). The apical domain is also the binding site for attachment proteins of pathogens, such as the haemorrhagic fever‐causing *Arenaviridae Machupo* virus glycoprotein 1, MGP1 (Radoshitzky et al., 2007; Sjöström, Berger, et al., 2020), and the malaria parasite *Plasmodium vivax* reticulocyte‐binding protein, PvRBP2b (Gruszczyk, Kanjee, et al., 2018), and possibly Rabies (Wang et al., 2023). Different protein engineering strategies have also been applied to develop protein binders that target the receptor apical by sheet extension (Sahtoe et al., 2021) and helical domains (Sjöström et al., 2022). The first one has a partially overlapping site with MGP1, while the latter overlaps with the HFE binding surface on the receptor. The reported values for the dissociation constants of some of the protein interactions with TfR are: 1–56 nM for Tf (Sjöström et al., 2022; West Jr. et al., 2000), 24–98 nM for HFE (Giannetti & Bjorkman, 2004; West Jr. et al., 2001), 9 nM for MGP1 (Sjostrom et al., 2023), and 20–400 nM for the sheet extension binders (Sahtoe et al., 2021). Similarly, the apical domain of different transferrin receptors has been demonstrated to have optimization potential as inhibitors of viral glycoproteins, among others, MGP1 (Cohen‐Dvashi et al., 2020; Sjostrom et al., 2023; Sjöström, Berger, et al., 2020).

Protein engineering has, over the last few decades, come to depend increasingly more on computational methodology. Advancements in machine learning, faster computers, DNA synthesis, and a better understanding of structure–function relationships have made protein design straightforward (Huang et al., 2016). Designing novel functions in protein scaffolds is still challenging with no easy approaches to computationally functionalize proteins concerning: (i) enzymes for reactions that have no known protein catalyst in nature, (ii) protein binders for any small hydrophilic molecules, and (iii) novel protein–protein interactions based on calculated absolute binding free energies. Common to these efforts is the complex nature of accurately calculating short‐ and long‐range polar interactions, which complicates the design of catalysts, binders, and protein–protein interfaces, as well as taking into account the intrinsically flexible nature of protein molecules. Water molecules, present in most biological functional interfaces, are often unaccounted for. Altogether, these result in difficult‐to‐predict tradeoffs between hydrogen bond formation, charge–charge interactions, and desolvation penalties of polar species.

The advantage of computational protein engineering strategies is often the ability to design towards a specific binding surface on the target of interest. This is also one of the strengths of the non‐antibody‐derived binder developments. Different protein scaffold topologies and protein biophysical properties, such as solvent‐accessible surface area (i.e., size) and charge distribution, allow for the exploration of a variety of characteristics. The cumulative accumulation of binders towards a target, in general, or as applied to the transferrin receptor system here, increases the number of engineered binders, with a higher likelihood that these may be effective. In the TfR case, it may help elucidate the best strategies for in vivo cellular delivery or the more challenging transport across the blood–brain barrier and as a blocking agent against pathogens targeting the apical domain. We have applied a method in which existing scaffolds in the PDB were repurposed solely based on the shape complementarity and surface area of the interaction with the target, without utilizing any knowledge of the naturally existing hotspot interactions. To find initial starting scaffolds for the design process, we screened a library consisting of approximately a thousand small proteins from the PDB directed towards the TfR apical domain. The protein interfaces with high buried surface area, surface complementarity, and total energy were thereafter designed. Besides allowing for design without prior knowledge of the interactions, the methodology enables the selection of specific protein topologies and minimizes the need to redesign the selected native topology. This contrasts with generative machine learning‐derived methods, which randomly hallucinate secondary structures and are often biased towards a high helical content. The obtained designs were characterized, and the best one was chosen for affinity maturation. Finally, we demonstrated binding both in vitro and in a cell‐based assay. The designed small protein, which targets the TfR apical domain, is relevant for medical applications, both as a starting point for the optimization of potential blocking therapeutics of *P. vivax* and *Arenaviridae* cell entry and as a carrier for cellular internalization by endocytosis.

## 2 RESULTS AND DISCUSSION

The transferrin receptor apical domain serves as an interaction target for both endogenous proteins, such as ferritin, and pathogens that cause hemorrhagic fevers or malaria. The binding to the apical domain, therefore, enables efficient crossing of the cell membrane for the delivery of iron or as part of the infectious process. We have engineered a binder towards the transferrin receptor apical domain for cellular delivery. Starting from a small protein scaffold set, initially superimposed onto the MGP1–apical domain complex, we carried out docking at the *Machupo* glycoprotein binding site. The best docked variants were chosen primarily based on interface size, which ranged from 600 to 1000 Å^2^. This resulted in 12 scaffolds for subsequent redesign rounds to improve binding. Out of these, transferrin receptor binding design TB14, based on the scaffold with PDB ID 2nzc (with a high buried interface area and good shape complementarity of the interface, Figure S1), showed the most promise and was affinity matured, characterized in vitro for binding to the receptor, and validated for cell delivery.

**FIGURE 1:**
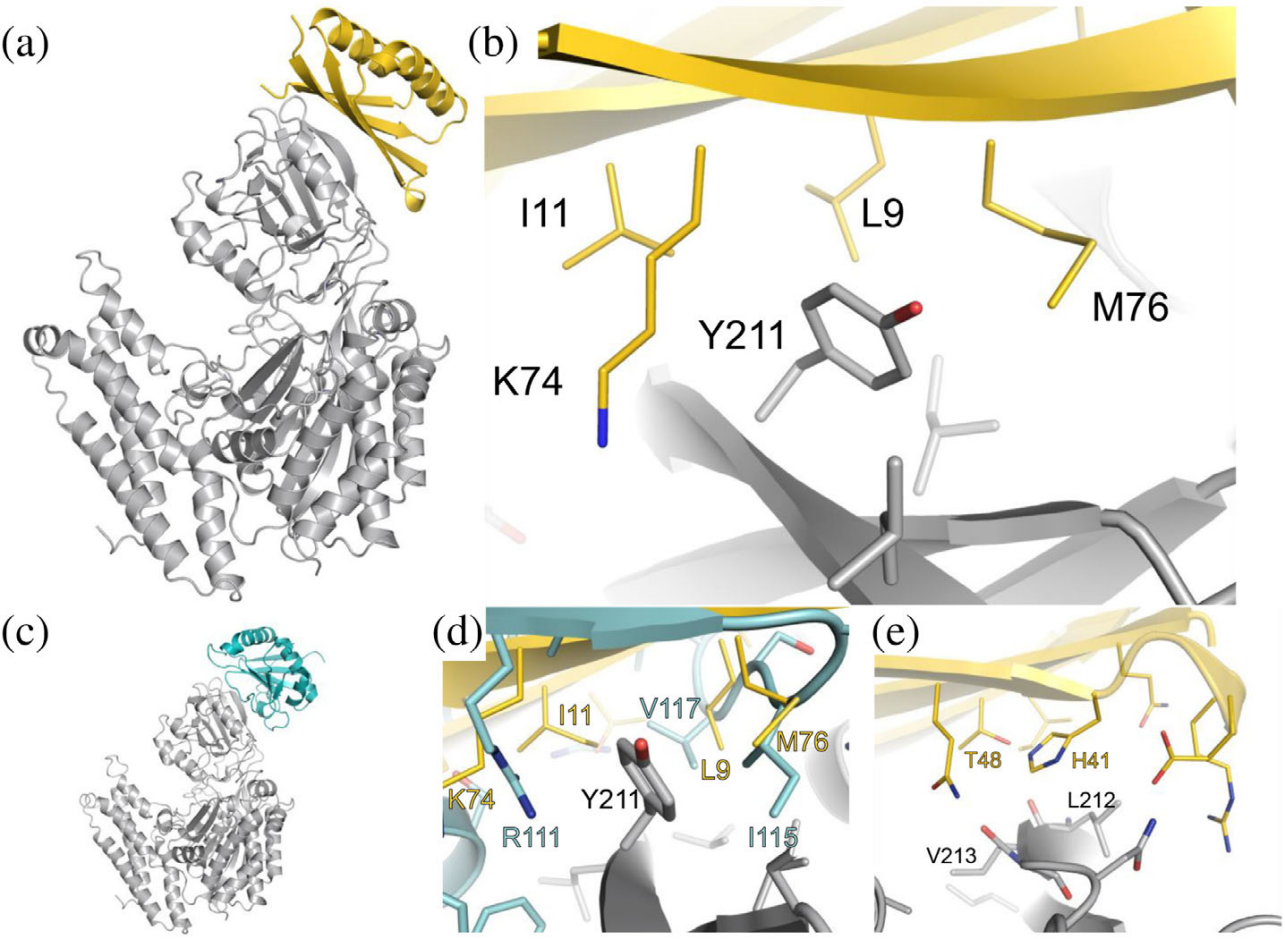
Computationally designed TB14–TfR interface and the comparison with MGP1–TfR. (a) The model of the designed TB14 variant (yellow) interaction interface with the apical domain of TfR. The secondary structure of the beta sheet follows the curvature of the apical domain. (b) A key element of the interface is the hydrophobic pocket created around TfR Tyr^211^. Mutations optimizing hydrophobic enveloping of TfR Tyr^211^: I7V, T9L, V11I, R74K, and K76M. (c) Machupo virus glycoprotein 1, MGP1, (teal) in complex with TfR (cyan) according to the experimental structure (PDB ID 3kas). (d) Hydrophobic packing comparison between TB14.2 (yellow) and MGP1 (cyan), surrounding TfR Tyr^211^. (e) The TB14.2 His^41^ in the TB14–TfR interface, which may be sensitive to intracellular pH‐dependent release from the receptor.

The protein–protein interface between TB14 and the receptor consists of four anti‐parallel β‐strands wrapping around the TfR apical domain (Figure 1a). It has been demonstrated experimentally by mutagenesis and experimentally determined structures of TfR in complex with MGP1, *Pv*RBP2b, and H‐ferritin that the Tyr^211^ receptor residue makes a crucial contribution to the formed interface (Abraham et al., 2010; Gruszczyk, Huang, et al., 2018; Montemiglio et al., 2019; Radoshitzky et al., 2011). In the designed protein, Tyr^211^ is surrounded by the hydrophobic residues Leu^9^, Ile^11^, and Met^76^, and the hydrophobic part of the Lys^74^ side chain (Figure 1b). The TB14 interface shows striking similarities with MGP1 regarding its hydrophobic packing around Tyr^211^ of TfR, when the crystal structure of MGP1–TfR (Figure 1c) PDB ID 3kas (Abraham et al., 2010) is overlaid with the designed interface of TB14–TfR: the methylene groups of MGP1 Arg^111^ and TB14 Lys^74^; MGP1 Val^117^ and TB14 Ile^11^ and Leu^9^; and, MGP1 Ile^115^ and TB14 Met^76^, respectively, surround the tyrosine hotspot (Figure 1d). Similarly, the malaria protein PvRBP2b, despite having a large interface with the TfR apical and protease‐like domains, interacts with the apical domain of the receptor at Tyr^211^ by surrounding it with two tyrosines and a serine residue (Gruszczyk, Huang, et al., 2018). Both the designed protein and the naturally occurring counterparts that interact with the apical domain appear to exploit the favorable interaction energetics associated with the receptor hotspot tyrosine. Interestingly, on the opposite side from Tyr^211^ of the TB14–TfR interface, TB14 His^41^ is at a possible hydrogen bonding donor‐acceptor distance of ~4 Å to the backbone oxygen of TfR Leu^212^ and Val^213^, respectively (Figure 1e). This may facilitate a pH‐dependent binding switch during receptor‐mediated transcytosis, potentially enabling intracellular release, although not investigated further here. Such a pH dependence has been exploited in histidine‐rich TfR‐binding antibodies, where a significant increase in cellular uptake was detected (Tillotson et al., 2015).

**FIGURE 2:**
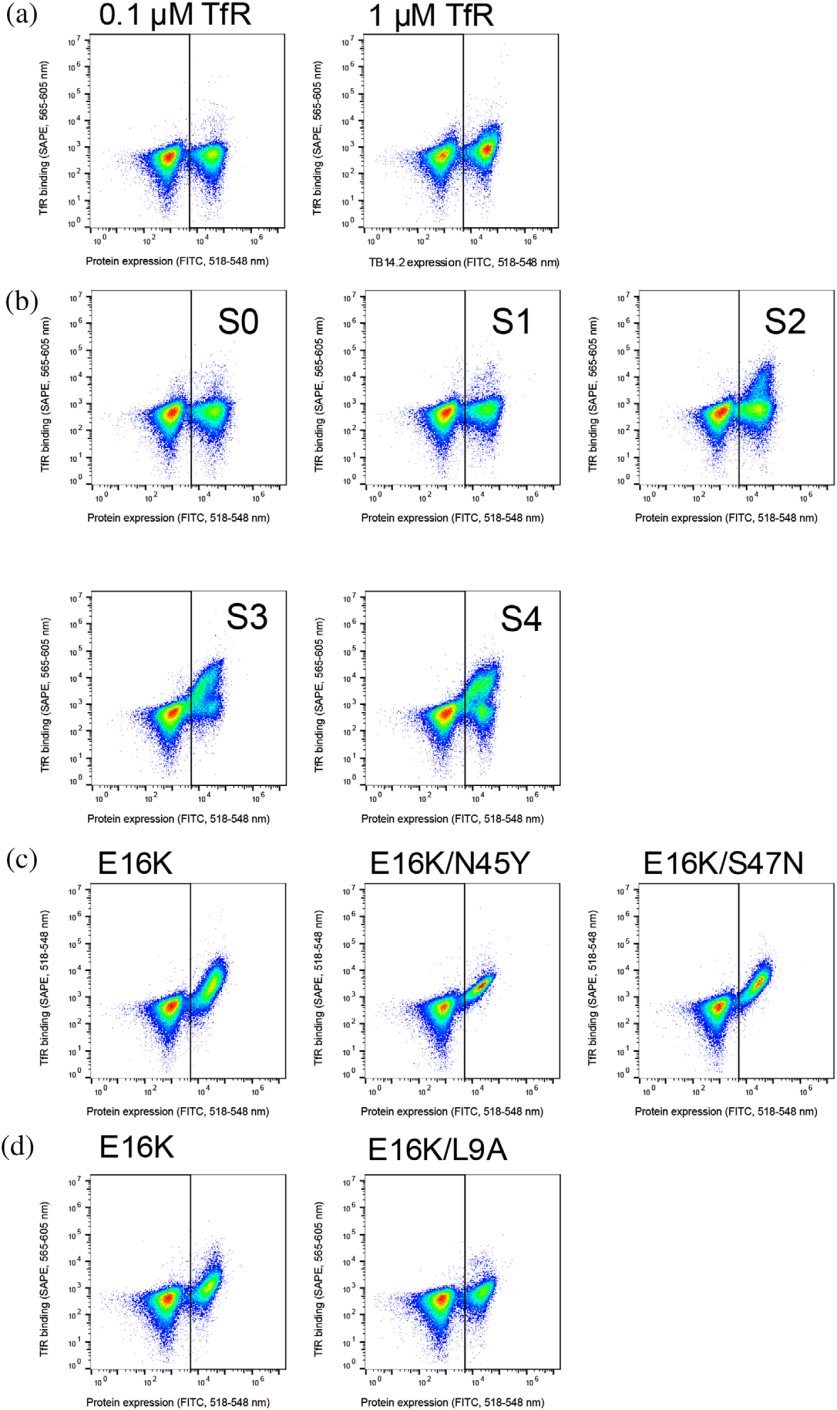
First round of directed evolution and analysis of the affinity‐matured variant TB14.2. Flow cytometry measurements were carried out for 50,000 singlet cells, where protein surface expression was monitored by anti‐c‐Myc FITC antibody binding signal (TB14.2 expression or its variants) while the formed complex was detected by SAPE bound to biotinylated TfR. (a) TB14.2 assayed at 100 nM and 1 μM TfR concentrations, respectively, shows an increase in the binding signal at the higher concentration. (b) The initial library and the four sorts from FACS assayed at 100 nM receptor concentration. (c) Three evolved mutants that were found during sorting (assayed at 100 nM TfR). (d) Comparison of the evolved variant E16K with the knockout mutation L9A implies the interaction interface formed as designed.

**FIGURE 3:**
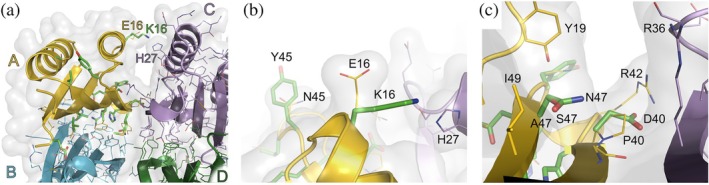
FACS selected mutations in TB14.2. (a) Residues introduced in TB14.2, compared to the scaffold protein (from *Thermotoga maritima*, PDB ID 2nzc, in homotetrameric form), and Lys^16^ are shown as green sticks on chain A (yellow). The evolved mutation E16K is far from the interface with TfR, but near the interface between chain A and chain C of the scaffold tetramer. (b) N45Y showed increased binding when combined with E16K, compared to solely E16K. Tyr^45^ is far from the TfR–TB14 interface, but near Lys^16^. (c) The second mutation that, in combination with E16K, further increases TfR binding is S47N (Ala^47^ in the scaffold) close to the interface with chain C.

The genes for the designed variants were transformed into a *S. cerevisiae* strain for the YSD binding assay by flow cytometry. The designed protein, TB14.2 (Figure S2), showed a 2‐fold higher binding signal at 1 μM TfR compared to 100 nM TfR (Figure 2a and Table S1). With error‐prone mutagenesis, a library of 3 × 10^5^ TB14.2 variants with (an average of 2 mutations per gene) was generated and sorted four times by FACS: at 1 μM TfR during the first two sorts and 100 nM TfR for the last two sorts (all sorts are assayed at 100 nM TfR in Figure 2b). Variants in each sorting step were sequenced, and during the second sort, E16K, E16K/S47N, and E16K/N45Y mutants were identified to bind TfR more strongly as measured by flow cytometry assay at 100 nM TfR (Figure 2c). The focused TB14.2 library contained only single or double mutations, which included E16K or a single E13K amino acid change (Table S2). The initial design showed no binding at this concentration, whereas E16K, E16K/N45Y, and E16K/S47N exhibited distinct binding signals, ranging from 7 to 11 times the background (Table S1). To determine if the E16K mutant is binding to the apical domain of TfR as designed, the knockout mutation L9A was introduced into the E16K variant. The E16K/L9A variant showed a lower binding signal for TfR than E16K alone, indicating that L9 is situated at the interface (Figure 2d). The experimentally selected variants during YSD/FACS contained mutations Tyr^45^ and Asn^47^ that are not near the TB14–TfR interface. Tyr^45^ is facing the solvent on a loop near Lys^16^, and Asn^47^ is close to the native scaffold oligomer interface between chain A and chain C (Figure 3a,b). Most of the mutations introduced in the scaffold during the computational design process, compared to the resulting protein variant TB14.2, are, as expected, located on the beta sheet surface in contact with the receptor (Figure 1a). The crystal structure of the scaffold protein from *Thermotoga maritima* (Cuff et al., 2006) forms a tetramer; therefore, the mutation P40D was introduced to prevent oligomerization from the non‐TfR interacting scaffold oligomerization interface (Figure 3c). By examining the position of the directed evolution mutation at the edge of the non‐TfR binding oligomerization interface, where the Lys^16^ nitrogen is 3.2 Å from the closest His^27^ nitrogen, E16K probably affects the oligomerization state of the design (Figure 3a). The size exclusion chromatogram also indicated a shift to a smaller size for the TB14.2 E16K (TB14.3) variant according to the elution volume (Figure S3). Interaction between the dimers may hinder binding to TfR; for example, when the scaffold tetramer is overlaid with the TB14–TfR structure, the TfR protease‐like domain and the second subunit are overlapping.

**FIGURE 4:**
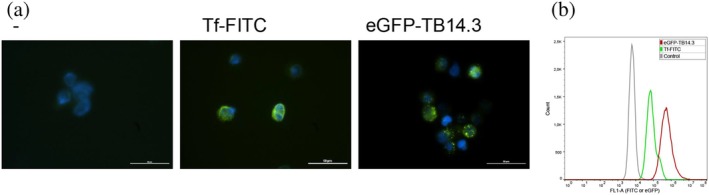
In vitro‐based assay of HeLa labeled cells. (a) HeLa cells incubated for 2.5 h with PBS (left), 160 nM Tf‐FITC (middle), or 640 nM eGFP‐TB14.3 (right) were detected with fluorescence microscopy. The reference bar indicates 50 μm. (b) Flow cytometry assay of HeLa labeled cells indicated binding of Tf and the designed protein, TB14.3, in comparison to the unlabeled control.

To further validate the binding interaction between the receptor and the designed protein, a stand‐alone receptor apical domain, AP01, was expressed (Sjostrom et al., 2020) and included in a competitive assay with TfR at a 100 nM concentration. The competition assay was carried out at 1 μM AP01 for the TB14.2 E16K/S47N variant or the mutant library, sorted three times (S3), and expressed on the yeast surface (Figure S4). The presence of a 10 times higher concentration of unlabeled, apical domain versus TfR significantly lowered the binding signal. The ability of the stand‐alone apical domain to compete with the receptor for binding to TB14.3 S47N further supports the binding of the designed protein to the TfR apical domain. The binding of eGFP‐tagged TB14.3 bacterially expressed protein was tested in vitro with human HeLa cells. HeLa cells were chosen for the experiments because, in many cancer cells, TfR expression is upregulated (Shen et al., 2018), including cervical cancer (Xu et al., 2019). As a positive control, the Tf protein chemically coupled with FITC fluorescent reporter was used. The results show that both Tf–FITC and eGFP–TB14.3 have a strong fluorescent signal according to fluorescent microscopy (Figure 4a) and measured by flow cytometry (Figure 4b). The GFP‐tagged native scaffold protein, eGFP–2nzc, was evaluated in comparison with eGFP–TB14.3 and Tf–FITC at 100 nM. The scaffold signal was just above the negative buffer control and clearly below the designed variant, which demonstrated a 7 times stronger signal, while transferrin was about 11 times better than the scaffold after subtracting the buffer signal (Figure S5). The GFP‐tagged designed protein supports binding to TfR in the HeLa cell assay.

**FIGURE 5:**
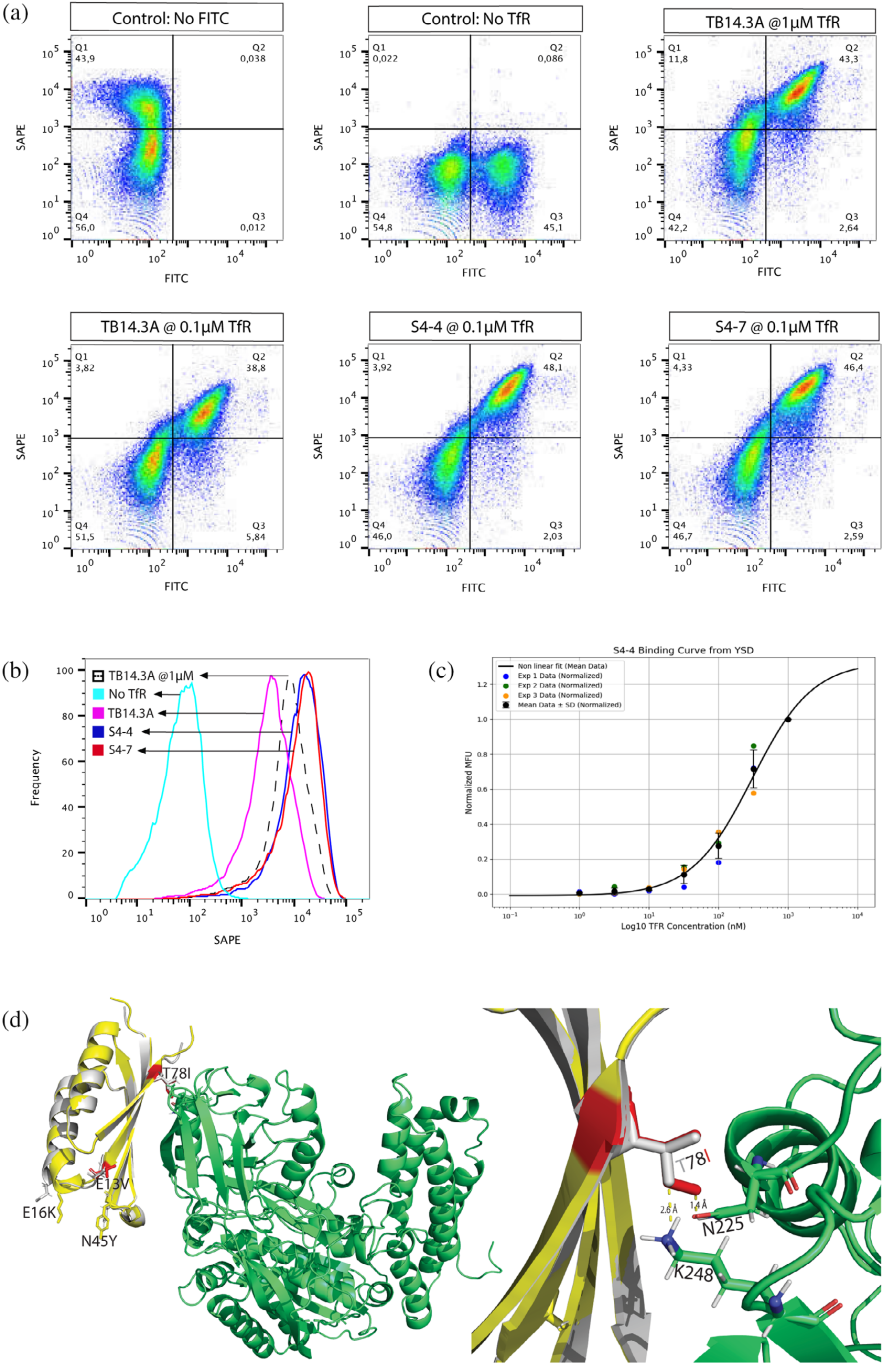
Second round directed evolution. (a) YSD of TB14.3A, evolved variants S4‐4, and S4‐7 at 0.1 μM TfR and controls at 1 μM TfR, events recorded for 50,000 singlets showing formed complex with TfR in Q2. (b) Comparison of binding signal from all expressing yeast cells among the different binders, showing a ten‐fold higher signal with TfR for S4‐4 and S4‐7 over TB14.3A. (c) Binding curve of S4‐4 variant determined from YSD plotted with varied TfR concentration and normalized MFU fitted non‐linearly and averaged from three experiments gave a *K*_d_ value of 305 ± 55 nM. (d) S4‐7 (yellow) superimposed with TB14.2 (gray) binding with TfR (green), showing all mutations as sticks (left panel). Position of round two mutation T78I and E13V marked in red. The common mutation in S4‐4 and S4‐7, T78I, shows proximity to the Asn^225^ and Lys^248^ residues in the receptor. The non‐polar carbon atom of isoleucine is positioned too close, at 1.4 Å, to the carbonyl oxygen of Asn^225^ in the original model, indicating some necessary rearrangement. Proximity to the sidechain methylene group of Lys^248^ at 2.6 Å supports a near‐favorable hydrophobic contact.

**FIGURE 6:**
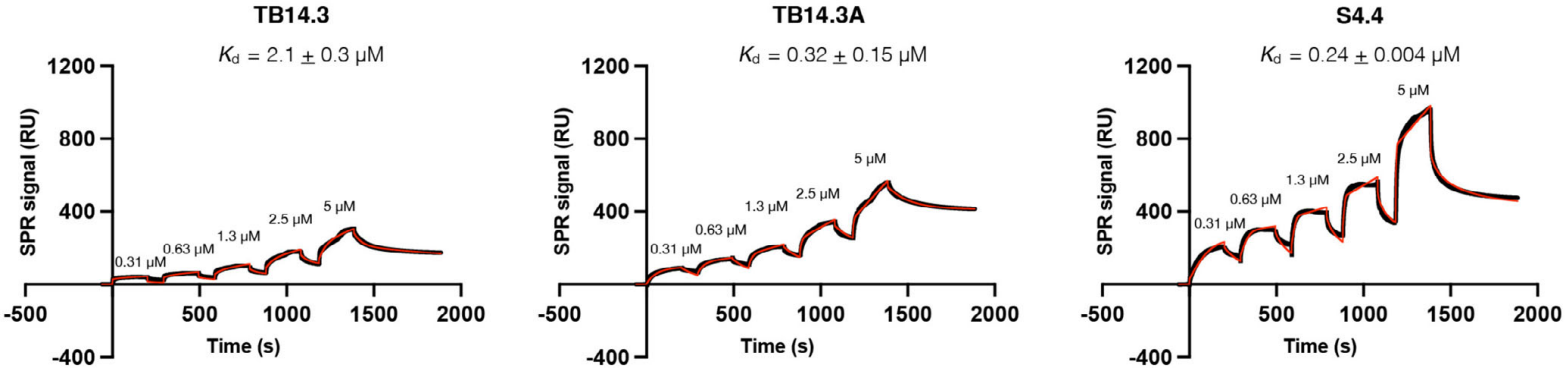
Surface plasmon resonance (SPR) measurements of TB14.3 and its variants, TB14.3A and S4.4, with TfR immobilized on the CM5 sensor chip by amine coupling approach. Single‐cycle kinetics were carried out to determine the dissociation constants (*K*_d_). The average and standard deviation of *K*_d_ values of two independent experiments are reported. The red curve represents the theoretical fit to the experimental data shown in black.

An effort to further improve the binding of TB14.2 E16K/N45Y (TB14.3A) was done by a second round of directed evolution following the protocol for TB14.2, resulting in a library having ~10^6^ variants with an average of 1–2 mutations per fragment. Four rounds of FACS, with the first two at 1 μM TfR gating for the top 5% of binders followed by 100 nM TfR gated for the top 1% of the binders (Figure S6), gave two better binding variants, TB14.3A T78I and TB14.3A E13V/T78I (Figure 5a,b), a single mutant (S4.4) and a double mutant (S4.7), respectively. Compared to the earlier variants, there is a shift towards the monomeric state during directed evolution rounds (Figure S3). The titration of the S4.4 variant by the YSD assay resulted in an apparent *K*_d_ value of 305 ± 55 nM (Figure 5c). Both S4.4 and S4.7 variants have a common mutation, T78I, which is located at the beta‐sheet interface of TB14 interacting with TfR Asn^225^ and Lys^248^ (Figure 5d). The strong binding signals observed for the evolved variants S4.4 and S4.7 in the flow cytometry assay (Figure 5b) were corroborated by surface plasmon resonance (SPR) measurements, which yielded dissociation constants (*K*_d_) in the high nanomolar range—specifically, *K*_d_ = 240 nM for S4.4 and *K*_d_ = 320 nM for TB14.3A (Figure 6), consistent with values obtained from the yeast‐based binding assay. Notably, the SPR analysis demonstrated an improvement in binding affinity for the second‐round evolved variants relative to the earlier variant TB14.3, which exhibited a significantly weaker affinity (*K*_d_ = 2 μM). Achieving the strongest possible binding to the TfR may not be beneficial for medical applications as demonstrated by the published work on TfR‐mediated transport across the blood–brain barrier (Yu et al., 2011). Antibodies with an IC50 of 111 nM for TfR resulted in the highest brain uptake. Following that hypothesis, we decided to stop evolving our library as we obtained a binder with a dissociation constant in the middle of the nM range.

## 3 CONCLUSIONS

We have demonstrated that the evolved, designed TB14 protein variants bind to the apical domain of TfR, as assayed by both the yeast surface display method, in vitro HeLa cell‐based assay, and SPR measurements. The most optimized variants represent valuable molecular tools for advancing our understanding of TfR‐mediated translocation mechanisms across cellular membranes. Furthermore, these variants hold potential as high‐affinity inhibitors capable of blocking the cellular entry of pathogenic agents that exploit the apical domain of TfR for host cell invasion. Additional rounds of affinity maturation may therefore be required to achieve the low nanomolar binding potential of both the natural and developed binders, as the best developed variants have dissociation constants of around 200 nM. The dual applicability underscores the utility of our variants both in fundamental research on receptor‐mediated endocytosis and in the development of targeted therapeutic strategies against receptor‐dependent pathogens.

## 4 MATERIALS AND METHODS

### 4.1 Computational design of receptor binders

Starting scaffolds for protein design were sorted out from the downloaded protein data bank (PDB) structures by fulfilling the following criteria: (a) length of 40–120 amino acids, (b) minimum of 1 α‐helix, (c) resolution lower than 3.0 Å, (d) R‐factor lower than 1.0, (e) no nucleic acids bound, (f) <99% sequence homology to the other included proteins, and (g) with non‐canonical amino acids converted to their closest canonical form. This resulted in approximately 1000 scaffolds. The proteins were aligned to the *Machupo* virus glycoprotein 1 (MGP1) in the holo crystal structure of the MGP1–TfR complex with PDB ID 3kas (Abraham et al., 2010). The aligned protein structures were optimized for interaction using the RosettaDock (Chaudhury et al., 2011) application by applying rigid body perturbation of up to 3.0 Å and rigid body rotation of up to 8.0 degrees. Each docking run generated 50 decoys for each starting complex. The 50 highest scoring interfaces based on buried surface area (BSA) and shape complementarity (SC) (Lawrence & Colman, 1993) were docked again with rigid body perturbation of up to 1.0 Å and rigid body rotation of up to 1.0° to achieve finer sampling of the interface. To optimize binding of the docked complexes, the interface residues were designed using the RosettaScripts (Fleishman, Leaver‐Fay, et al., 2011) application. The design methodology was followed according to the published protocol in Sjöström et al (Sjöström et al., 2022). The best‐designed protein, TB14, based on the PDB ID 2nzc from *Thermotoga maritima*, was chosen for experimental characterization. Two additional variants of TB14 were designed: TB14.1 (containing D32Y, T55V and T59A mutations) and TB14.2 (D32Y, P40D, T55V, and T59A), relative to TB14, respectively (for the TB14.2 sequence cf. Figure S2).

### 4.2 Cloning and plasmid purification

The linear pETCON2 (Fleishman, Whitehead, et al., 2011) plasmid, digested with FastDigest *Nde*I and *Xho*I (Thermo Scientific, USA), and a corresponding gene, was used to construct the expression vectors by homologous recombination in the *Saccharomyces cerevisiae* EBY100 strain. The genes encoding designs were ordered from IDT (Integrated DNA Technologies, USA) with 50–60 nucleotide overhangs that corresponded to the pETCON2 vector before or after the *Nde*I/*Xho*I restriction sites, respectively. The transformation of yeast cells was performed by the heat‐shock procedure described in Gietz and Schiestl (32) with selection on agar plates made with C–UT growth medium. The C–UT medium was prepared as follows: 1.85 g/L synthetic complete mixture, Kaiser, drop‐out –Trp –Ura (Formedium, England), 6.9 g/L yeast nitrogen base without amino acids with ammonium sulphate (Formedium, England), and 20 g/L d–(+)–glucose (Sigma–Aldrich, USA).

The cloning was confirmed by sequencing after colony PCR. Each colony was dissolved in 20 μL reactions containing 5 mg/mL Zymolase (Seikagaku Corporation, Japan), 250 mM HEPES, 2 M sorbitol, and 15% glycerol, and incubated for 1 h at 37°C in adaptation of the protocol from Singh et al (Singh & Weil, 2002). 2 μL of the zymolase reaction was subjected to 30 PCR amplification cycles with the forward and reverse primers: CCATACGACGTTCCAGACTACG and CTATTACAAGTCCTCTTCAGAA. After PCR cleanup with ExoSAP‐IT (ThermoFisher Scientific, USA), one of the primers was added to the Mix2Seq kit (EuroFins, Germany) for sequencing. For plasmid purification, cells were lysed under the zymolase reaction, from 10 mL of yeast cells, grown ON to an OD_600_ = 6 in C–UT growth medium. The plasmid was purified from yeast according to the QIAprep Miniprep Kit (Qiagen, Germany).

### 4.3 Flow cytometry analysis

The yeast surface display (YSD) method (Chao et al., 2006) was used to assay the binding of designed variants to TfR experimentally. With YSD, we monitored both the expression of the designed proteins on the yeast cell surface and the binding to the target receptor. For the binding assay, the avi‐tagged TfR was expressed and purified from HEK 293F cells and subsequently biotinylated according to the protocol in Sjöström et al. (Sjöström, Lundgren, et al., 2021). EBY100 yeast cells, with the recombinant pETCON2 vector, were passaged overnight in C–UT medium with glucose and centrifuged at 2500 × *g* for 5 min. Protein expression was induced by resuspending the cells in C–UT medium containing galactose instead of glucose, at OD_600_ = 0.5, and incubating at 20°C for 20 h, shaking at 180 rpm. 50 μL induced cells at OD_600_ = 1 were washed with PBSF (8 g NaCl, 0.2 g KCl, 1.44 g Na_2_HPO_4_, 0.24 g KH_2_PO_4_, and 1 g bovine serum albumin in 1 L deionized sterile filtered H_2_O, adjusted to pH 7.4), and incubated with 1 μM TfR for 90 min on ice, in 50 μL. Alternatively, for lower concentrations of TfR, in a volume resulting in at least 10 times more TfR than the approximate number of surface‐expressed TB14 molecules. After another wash with PBSF, the cells were labeled on ice, in the dark for 30 min in 50 μL with 1:100 diluted chicken anti‐C‐myc FITC conjugated antibody (Immunology Consultants Laboratory), which monitored yeast surface protein expression, and 1:19 SAPE (Streptavidin–R‐Phycoerythrin Conjugate; Invitrogen, USA), which detected biotinylated TfR binding to yeast expressed protein, in PBSF. The cells were washed with PBSF and spun down, before dissolving the cell pellet in PBSF and measuring 50,000 single yeast cells on a BD Accuri C6 flow cytometer (a blue 488 nm laser for excitation and the bandpass filters 533/30 and 585/40 for detecting FITC and SAPE, respectively). Flow cytometry figures were prepared with FlowJo™ software (2019). The labeling and binding protocol was followed in the competition assay with 1 μM unlabeled apical domain, AP01 (Sjostrom et al., 2020), assaying variant TB14.2 E16K/S47N and library sort 3 at 100 nM TfR. AP01 was purified following the published protocol in Sjöström et al (Sjostrom et al., 2020).

### 4.4 Construction of a mutagenesis library

The pETCON2 plasmid containing the variant insert was used for random mutagenesis (Mutazyme II kit, Stratagene, USA), with the same primers as for sequencing, to generate a library of TB14.2 variants, followed by the second round of mutagenesis on the best variant TB14.3A (TB14.2 E16K/N45Y). Two reactions were set up with 5 ng and 500 ng of the total amount of gene in the plasmid to titrate the optimal number of mutations per gene. Both PCR reactions were transformed into yeast by electroporation in 0.2 cm cuvettes (Bio‐Rad, USA), with 1 μg pETCON2, 3 μg randomized fragments in a 100 μL volume, following the published protocol (Benatuil et al., 2010), with 0.1 M LiAc (Sigma‐Aldrich, USA), and 10 mM DTT (Fisher Bioreagents, USA) for conditioning of cells. The electroporated cells were transferred into 10 mL of a 1:1 solution of 2 M sorbitol and YPD medium (20 g/L peptone (Nordic biolabs, Sweden), 10 g/L yeast extract (Sigma‐Aldrich, USA), 20 g/L d‐(+)‐glucose (Sigma‐Aldrich, USA)), and incubated at 30°C at 180 rpm shaking for 1 h. The cells were collected and resuspended in 50 mL C–UT medium, and the number of transformants was determined by plating 5 and 50 μL of the culture on selective C–UT agar plates. After incubation at 30°C for 2 days, colony‐forming units were counted to calculate the size of the mutagenesis library. The number of mutations per gene was determined based on DNA sequencing using the Mix2Seq kit (EuroFins, Germany).

### 4.5 Fluorescence‐activated cell sorting of the variants

EBY100 yeast cells were passaged overnight in C–UT medium containing glucose, and protein surface expression was induced by switching to C–UT medium containing galactose, incubating at 20°C with 180 rpm shaking. 500 μL induced cells OD_600_ = 0.5 were pelleted, washed with 1 mL PBSF, labeled on ice for 1.5 h at 1 μM TfR in 500 μL with PBSF. The first two sorts were labeled with 1.0 μM TfR and the last two with 100 nM TfR. Centrifugation was done with a tabletop centrifuge at 2500 × *g* for 5 min. With samples kept on ice until measurement, another wash with 1 mL PBSF was performed before 30 min of labeling with 1:100 diluted chicken anti‐C‐myc‐FITC conjugated antibody (Immunology Consultants Laboratory, USA) and 1:19 SAPE, reaching a total volume of 500 μL of reaction in PBSF. The cells were washed with PBSF, pelleted, and kept on ice until sorting with a BD Influx for the first two sorts and a Biorad S3e for the last two sorts of the first round of directed evolution. The second round of directed evolution was carried out on a BD Melody cell sorter. For the BD Influx and Melody, SAPE was excited with a yellow‐green laser (561 nm) and FITC with a blue laser (488 nm), with the bandpass filters in BD Influx used to detect SAPE and FITC being 585/29 and 530/40, and for BD Melody being 582/15 and 527/32, respectively. The excitation wavelength for the Biorad S3e assay was 488 nm, and the bandpass filters used for FITC and SAPE were 525/30 and 586/25 nm. Cells were resuspended in PBSF, gated on the top 5% in the first sort and top 1% of cells in subsequent rounds of FACS, in a triangular gate on the diagonal top left of the FITC positive population. Cells were collected in C–UT supplemented with penicillin and streptomycin. Between each round of FACS, the cells were passaged twice in 50 mL C–UT medium.

### 4.6 Protein expression and purification

The gene encoding TB14.2 E16K (TB14.3) and its sort variants were amplified out from pETCON2. 2nzc and TB14.3 C‐terminally fused to enhanced green fluorescent protein (eGFP) were synthesized by IDT (Integrated DNA Technologies, USA) (Figure S2). The variants were cloned into the pET29b(+) plasmid at *Nde*I/*Xho*I restriction sites with FastDigest enzymes (Thermo Scientific, USA). Tuner cells were heat‐shock transformed with the respective variant vector and plated onto LB agar plates supplemented with kanamycin. Colony PCR was carried out with T7 primers to confirm the cloned gene by sequencing. The variants were grown in LB media until OD_600_ = 0.6; protein expression was induced by 1 mM IPTG at 20°C for 18 h. Cells were pelleted at 8000 × *g* for 10 min at 4°C, resuspended in purification buffer (25 mM HEPES, 100 mM NaCl, pH = 7.4), and sonicated on ice four times at 40% amplitude, 20 s on 20 s off, with a Vibra Cell 100 sonicator. 2 mL 50% Cobalt resin (Thermo Scientific, USA) was loaded onto a gravity column (Thermo Scientific, USA), and equilibrated with 5 bed volumes of the purification buffer. The sonicated cells were centrifuged at 15,000 × *g* for 30 min at 4°C; the supernatant was passed with a syringe through a 0.2 μm filter before loading onto the column. The wash step was done with 5 bed volumes of purification buffer supplemented with 10 mM imidazole and eluted with purification buffer supplemented with 500 mM imidazole. The best elutions, confirmed by SDS‐PAGE, were pooled and additionally purified by size exclusion chromatography (SEC) on an ÄKTAgo FPLC system over Superdex 200 Increase 10/300 or HiLoad 16/60 Sephacryl S‐100 columns with the purification buffer. Fractions corresponding to the protein according to the *A*_280_ signal were collected and assayed by SDS‐PAGE.

### 4.7 HeLa cell culture, flow cytometry, and microscopy analysis

Human epithelial HeLa cell line was cultured in Dulbecco's modified Eagle's medium (DMEM) supplemented with 10% fetal bovine serum, 2 mM L‐glutamine, 100 U/mL penicillin, and 100 μg/mL streptomycin at 37°C under a humidified 5% CO_2_ atmosphere and subcultured when confluent (chemicals from Sigma–Aldrich). HeLa cells were seeded on six‐well plates, 4 × 10^6^ cells/well, and allowed to grow for 48 h; the aforementioned cell culture medium and conditions were used. Human holo‐Tf (T0665, Sigma–Aldrich, USA) was labeled with the Pierce FITC Antibody Labeling Kit (Thermo Scientific, USA), according to the manufacturer's standard protocol, which resulted in a labeling ratio of 6:1 of FITC:Tf. The culture medium was removed, cells were rinsed twice with phosphate‐buffered saline (PBS, Sigma–Aldrich), and the confluent cells were incubated with Tf–FITC and eGFP‐TB14.3 in PBS at final protein concentrations of 160 and 640 nM, respectively, for 2.5 h. Cells in PBS were used as a negative control. Plates with cells were rotated three times during the labeling process. HeLa cells were mechanically detached from the plate and washed twice (100 g, 2 min) with PBS before flow cytometry measurement (BD Accuri C6), using a 480 nm blue laser for excitation and 525/30 filter for detection. Flow cytometry figures were prepared with FlowJo software (2019). For the control experiment, eGFP–TB14.3, the native scaffold eGFP–2nzc, and Tf–FITC were assayed at 100 nM on BD Melody following the protocol above. Before the microscopy analysis, labeled HeLa cells (10 μL) were mounted with anti‐fading mounting medium Vectashield containing 4',6‐diamidino‐2‐phenylindole, 2HCl (DAPI, Vector Laboratories, Burlingame, CA, USA). A Nikon epifluorescence microscope (Nikon, Tokyo, Japan) equipped with appropriate filters was used. Images were acquired with a digital camera acquisition system (Nikon, DS‐U1) by using the software Nis‐element imaging, version 4.3, Nikon.

### 4.8 Surface Plasmon resonance (SPR) measurements

Biacore T200 instrument (Cytiva) was used to perform Surface Plasmon Resonance (SPR) experiments. Recombinant TfR (R&D Systems) was immobilized by amine‐coupling reactions on a CM5 sensor chip (Cytiva), according to the manufacturer's instructions, and reached an immobilization level of 1100 RU. To determine the dissociation equilibrium constant (*K*_d_), TB14.3 and its variants, TB14.3A and S4.4, were injected, using a single cycle kinetic mode with an association phase of 240 s and a dissociation phase of 500 s. HBS‐P+ (HEPES 10 mM, NaCl 150 mM, p20 0.05%) (Cytiva) was used as the running buffer. The dissociation constants (*K*_d_) were determined using the BIAEVALUATION 3.0 software, using a two‐exponential equation, which better describes the complexity of the binding. The protein concentrations tested were 0.31, 0.63, 1.3, 2.5, and 5 μM.

## AUTHOR CONTRIBUTIONS

**Anuthariq Alikkam Veetil:** Writing – review and editing; conceptualization; methodology; formal analysis; validation. **Dick J. Sjöström:** Conceptualization; methodology; validation; formal analysis. **Cristian Iribarren:** Validation. **Camilla Mohlin:** Validation; writing – review and editing. **Elena Ambrosetti:** Validation; writing – review and editing. **Sinisa Bjelic:** Conceptualization; methodology; validation; formal analysis; software; supervision; project administration; funding acquisition; resources; writing – original draft; writing – review and editing.

## Supporting information


**DATA S1.** Additional information relating to FACS sorting.

## Data Availability

The data that support the findings of this study are available from the corresponding author upon reasonable request.
