# Supplementary material for: Targeting the apical domain of the transferrin receptor: Development of a new protein scaffold for cellular delivery
Source: Protein Sci. 2025 Nov 18;34(12):e70384. doi: 10.1002/pro.70384 (PMC12626770; doi:10.1002/pro.70384)
Supplement: Supplementary file 1 — DATA S1. Additional information relating to FACS sorting. [file PRO-34-e70384-s001.pdf]

Supplementary Material

Targeting the Apical Domain of the Transferrin Receptor: Development of a New Protein Scaffold for Cellular Delivery

Anuthariq Alikkam Veetil, Dick J Sjöström, Cristian Iribarren, Camilla Mohlin, Elena Ambrosetti, & Sinisa Bjelic

Table S1. Normalized Mean fluorescent units for first round evolution.

The mean fluorescence units (MFU) of the protein expressing population subtracted with the protein expressing population without added TfR for first round evolution are listed. The TfR binding for sorts and for evolved TB14.2 mutations was assayed 100 nM receptor concentration.

**Sorts**	MFU Accuri	Norm. to TB14.2
TB14.2, 1 μM TfR	626	1.0
TB14.2	287	1.0
Sort 0	282	0.98
Sort 1	376	1.3
Sort 2	1290	4.5
Sort 3	4642	16.2
Sort 4	3652	12.7
**Evolved mutations**	MFU Accuri	Norm. to TB14.2
E16K	3004	10.5
E16K N45Y	2128	7.4
E16K S47N	3256	11.3
**Knockout mutation**	MFU Accuri	Norm. to E16K
E16K	1191	1.0
E16K L9A	529	0.44

Table S2. Identified mutations during FACS of the TB14.2 library.

Improved binding to TfR is indicated with a plus sign and stronger binding is indicated with two plus signs. Sort 1 and 2 carried out after labelling at 1 μM TfR, and sort 3-5 at 0.1 μM.

library unsorted-sort 5	number of sequences	% of all	TfR binding
**Unsorted**			
E13N/L51Q/N57D	1	14	
E13L/N45I/S84R	1	14	
V52G/L53M/S71I/G72C	1	14	
I10V/D58E	1	14	
R36H	1	14	
H41L	1	14	
Wt	1	14	
**S1**			
D40N/L53V	1	25	
H41Q	1	25	
E16K/Q39K	1	25	
N45K	1	25	
**S2**			
E16K	1	25	++
I11N/E16K	1	25	++
E16K/S47N	1	25	++
E16K/N45Y	1	25	++
**S3**			
E13K	5	50	++
E16K	3	30	++
E16K/Q21K	1	10	
E43K	1	10	
**S4**			
E16K	3	60	++
E16K/Q21K	1	20	
L9M/E16K	1	20	

Table S3 Normalized Mean fluorescent units for second round evolution.

The mean fluorescence units (MFU) of the protein expressing population subtracted with the protein expressing population without added TfR for all shown figures are listed. The TfR binding for sorts and for evolved TB14.3A mutations was assayed 100 nM receptor concentration.

**Variants**	MFU	Norm MFU
TB14.3A, 1 μM TfR	8271	1.0
TB14.3A	3720	0.45
Sort 0	4716	0.57
Sort 1	5374	0.65
Sort 2	4788	0.58
Sort 3	5725	0.69
Sort 4	6072	0.73
TB14.3A, 1 μM TfR	9867	1.0
TB14.3A	4485	0.45
S4-4	16300	1.65
S4-7	15646	1.58

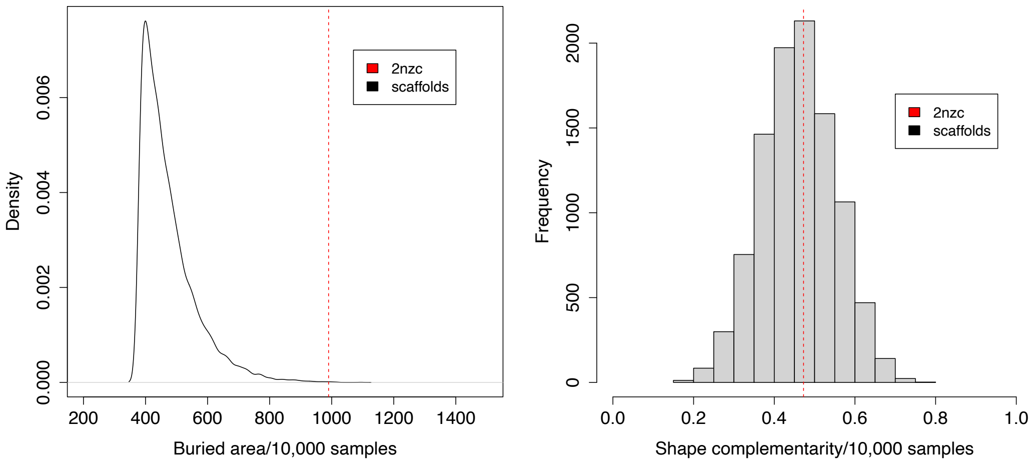

Figure S1. The 2nzc scaffold compared to other scaffolds used in the design. Buried are (left) and shape complementarity (right) of the TfR interface.

a)

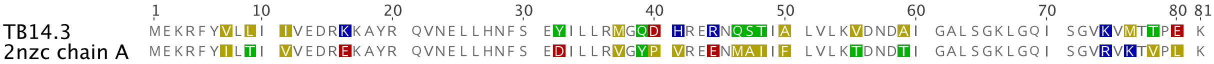

b)

TB14

ATGGAAAAACGCTTTTATGTGCTGCTGATTATTGTGGAAGATCGCGAAAAAGCGTATCGCCAGGTGAACGAACTGCTGCATAACTTTAGCGAAG
ATATTCTGCTGCGCATGGGCCAGCCGCATCGCGAACGCAACCAGAGCACCATTGCGCTGGTGCTGAAAACCGATAACGATACCATTGGCGCGCT
GAGCGGCAAACTGGGCCAGATTAGCGGCGTGAAAGTGATGACCACCCCGGAAAAACGCGGCAGCCTCGAG

TB14.1

CATATGGAAAAACGCTTTTATGTGCTGCTGATTATTGTGGAAGATCGCGAAAAAGCGTATCGCCAGGTGAACGAACTGCTGCATAACTTTAGCG
AATATATTCTGCTGCGCATGGGCCAGCCGCATCGCGAACGCAACCAGAGCACCATTGCGCTGGTGCTGAAAGTGGATAACGATGCGATTGGCGC
GCTGAGCGGCAAACTGGGCCAGATTAGCGGCGTGAAAGTGATGACCACCCCGGAAAAACGCGGCAGCCTCGAG

TB14.2

CATATGGAAAAACGCTTTTATGTGCTGCTGATTATTGTGGAAGATCGCGAAAAAGCGTATCGCCAGGTGAACGAACTGCTGCATAACTTTAGCG
AATATATTCTGCTGCGCATGGGCCAGGATCATCGCGAACGCAACCAGAGCACCATTGCGCTGGTGCTGAAAGTGGATAACGATGCGATTGGCGC
GCTGAGCGGCAAACTGGGCCAGATTAGCGGCGTGAAAGTGATGACCACCCCGGAAAAACGCGGCAGCCTCGAG

TB14.3

CATATGGAAAAACGCTTTTATGTGCTGCTGATTATTGTGGAAGATCGCAAAAAAGCGTATCGCCAGGTGAACGAACTGCTGCATAACTTTAGCG
AATATATTCTGCTGCGCATGGGCCAGGATCATCGCGAACGCAACCAGAGCACCATTGCGCTGGTGCTGAAAGTGGATAACGATGCGATTGGCGC
GCTGAGCGGCAAACTGGGCCAGATTAGCGGCGTGAAAGTGATGACCACCCCGGAAAAACGCGGCAGCCTCGAG

c)

CATATGGTGAGCAAAGGTGAAGAACTGTTTACCGGTGTTGTTCCGATTCTGGTTGAACTGGATGGTGATGTTAATGGCCACAAATTTTCAGTTA
GCGGTGAAGGCGAAGGTGATGCAACCTATGGTAAACTGACCCTGAAATTTATCTGTACCACCGGCAAACTGCCGGTTCCGTGGCCGACACTGGT
TACCACACTGACCTATGGTGTTCAGTGTTTTAGCCGTTATCCGGATCACATGAAACAGCACGATTTTTTCAAAAGCGCAATGCCGGAAGGTTAT
GTTCAAGAACGTACCATCTTCTTCAAAGATGACGGCAACTATAAAACCCGTGCCGAAGTTAAATTTGAAGGTGATACCCTGGTGAATCGCATTG
AACTGAAAGGCATCGATTTTAAAGAGGATGGTAATATCCTGGGCCACAAACTGGAATATAATTATAATAGCCACAACGTGTACATCATGGCCGA
CAAACAGAAAAATGGCATCAAAGTGAACTTCAAGATCCGCCATAATATTGAAGATGGTTCAGTTCAGCTGGCCGATCATTATCAGCAGAATACC
CCGATTGGTGATGGTCCGGTTCTGCTGCCGGATAATCATTATCTGAGCACCCAGAGCGCACTGAGCAAAGATCCGAATGAAAAACGTGATCACA
TGGTGCTGCTGGAATTTGTTACCGCAGCAGGTATTACCTTAGGTATGGATGAACTGTATAAAGGTGGTGGATCCAGCAGCATGGAAAAACGCTT
TTATGTGCTGCTGATTATTGTGGAAGATCGCAAAAAAGCGTATCGCCAGGTGAACGAACTGCTGCATAACTTTAGCGAATATATTCTGCTGCGC
ATGGGCCAGGATCATCGCGAACGCAACCAGAGCACCATTGCGCTGGTGCTGAAAGTGGATAACGATGCGATTGGCGCGCTGAGCGGCAAACTGG
GCCAGATTAGCGGCGTGAAAGTGATGACCACCCCGGAAAAACGCGGCAGCCTCGAG

Figure S2. TB14.3 (TB14.2 E16K) amino acid and gene sequence. a) Alignment of the TB14.3 amino acid sequence and the scaffold protein PDB ID: 2nzc. **b**) DNA sequence of TB14 variants, with *Nde*I and *Xho*I restriction cleavage sites included. **c)** DNA sequence of eGFP-TB14.3.

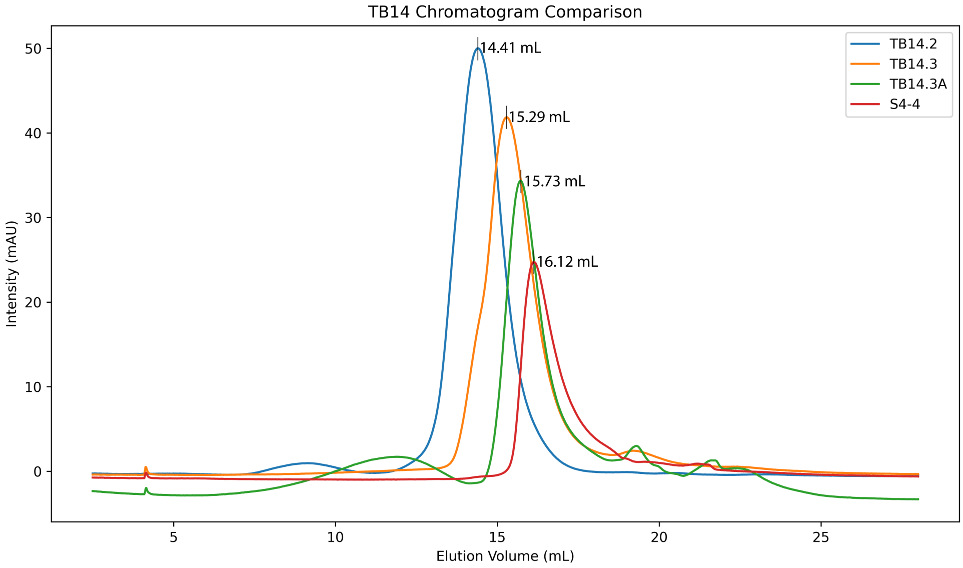

Figure S3. Size exclusion chromatogram of TB14.2 and the evolved variants shows a trend towards a smaller size according to the elution volumes.

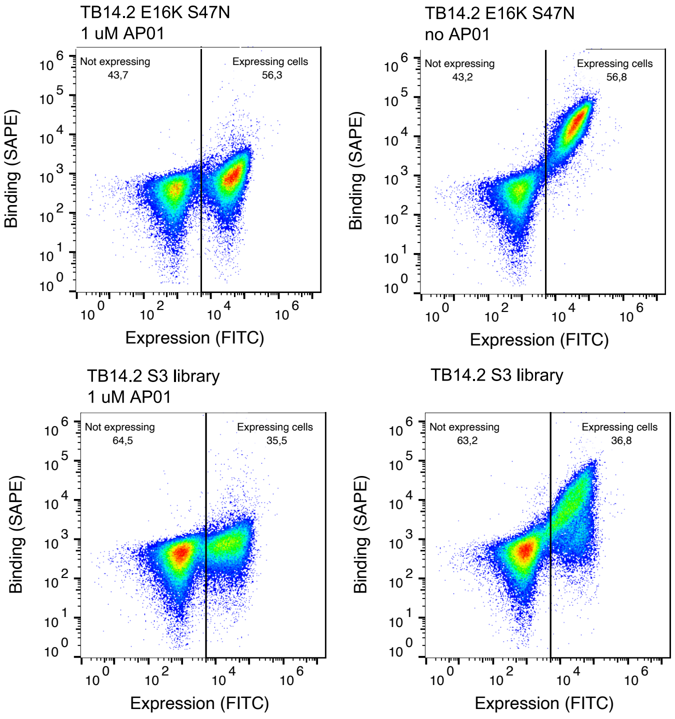

Figure S4. Competition of TB14.2 variants with unlabeled bacterially expressed TfR apical domain and TfR. Variants from the sorted library (S3) and the E16K/S47N mutant from S3 were labeled with 100 nM TfR. The first column was incubated additionally with 1 µM solubilized apical domain AP01 PDB ID: **6y7**. The standalone apical domain competes with TfR and indicates that the designed protein binds to the correct domain of the receptor.

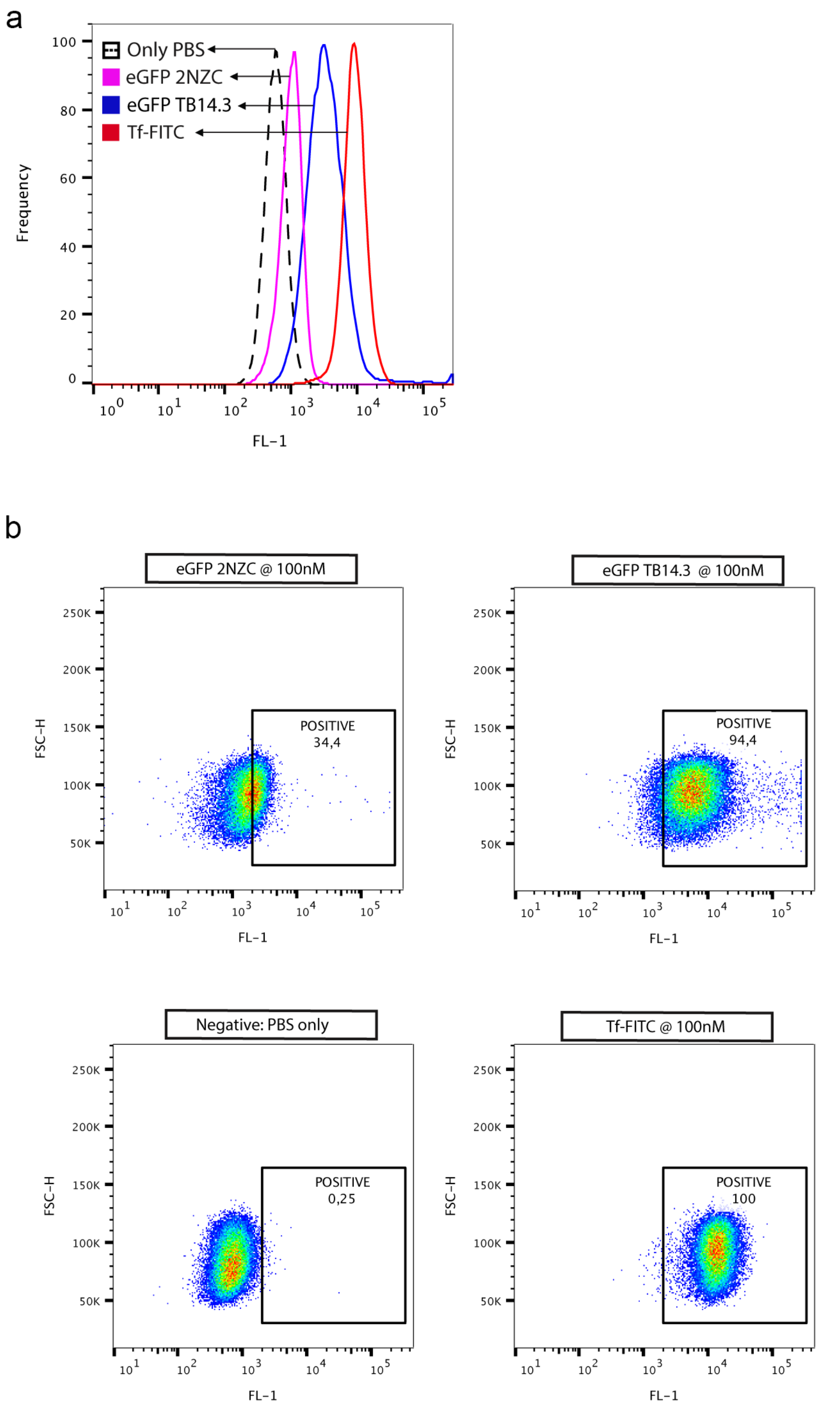

Figure S5. Evaluation of the fluorescently-tagged, native scaffold, eGFP–2NZC and the TfR designed binder, in the HeLa cell assay by flow cytometry. The binding signal for the scaffold was just above the buffer control, PBS, according to a) histogram and b) dot plots assayed at 100 nM. Tf–FITS was used as the positive control.

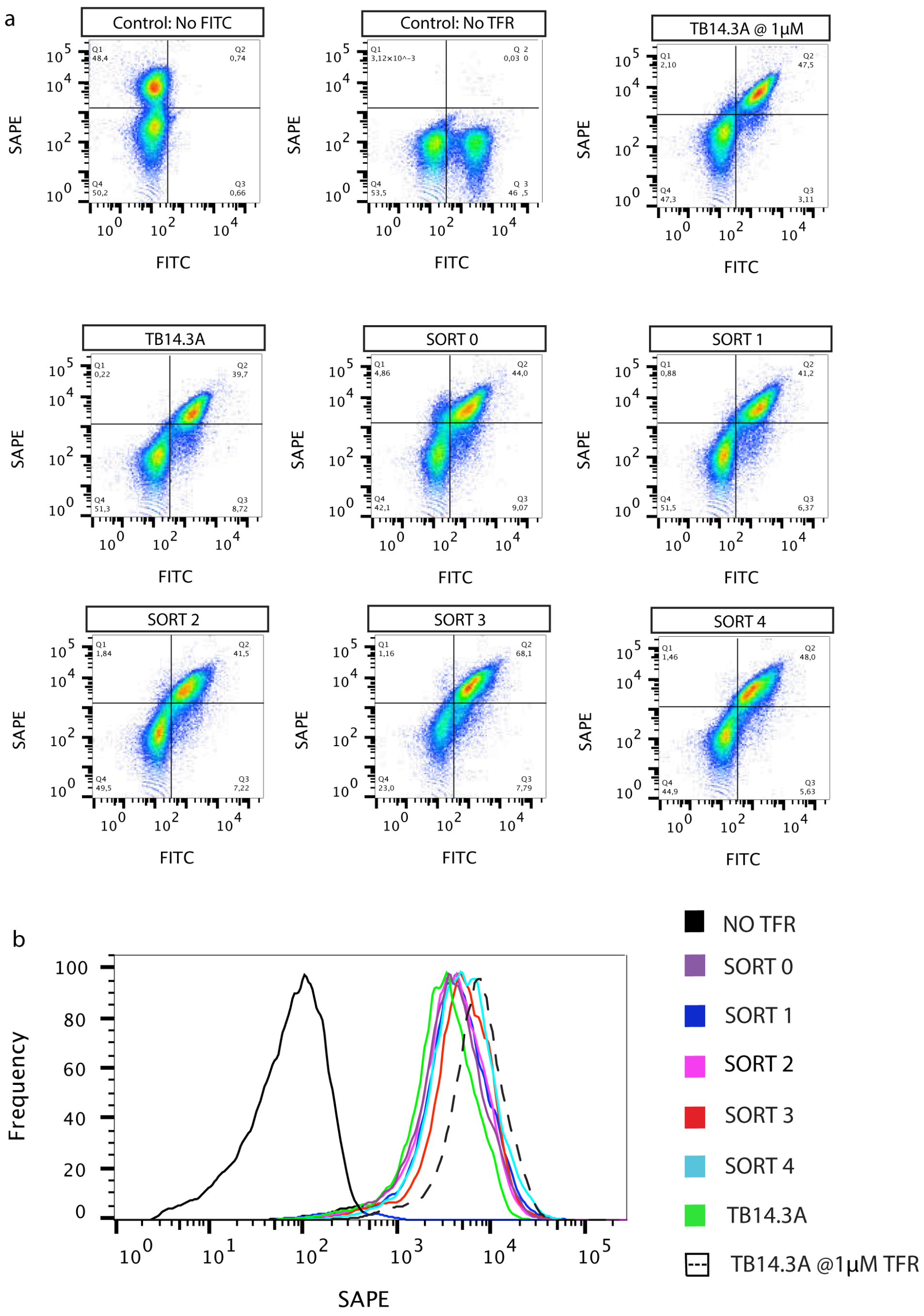

Figure S6. TB14.3A library sort progression. a) TB14.3A and its library sort 0 along with four consecutive sorted library showing binding signal with 0.1μM TfR where controls are incubated with 1μM TfR. where FITC signal gives the extend of expression and SAPE signal gives the extend of binding **b)** Comparison of binding signal from all expressing cells among the different library versions, showing higher signal for sort 4.
